# The physician’s experience of changing clinical practice: a struggle to unlearn

**DOI:** 10.1186/s13012-017-0555-2

**Published:** 2017-02-28

**Authors:** Divya M. Gupta, Richard J. Boland, David C. Aron

**Affiliations:** 1 School of Medicine, Case Western Reserve University, Cleveland, OH USA; 2 Weatherhead School of Management, Case Western Reserve University, Cleveland, OH USA; 3 Louis Stokes Cleveland Dept. of Veterans Affairs Medical Center, Admin Building (EUL) Room 5 M677, Cleveland, OH 44106 USA

**Keywords:** Unlearning, Practice change, Grounded theory, Qualitative methods

## Abstract

**Background:**

Changing clinical practice is a difficult process, best illustrated by the time lag between evidence and use in practice and the extensive use of low-value care. Existing models mostly focus on the barriers to learning and implementing new knowledge. Changing clinical practice, however, includes not only the learning of new practices but also unlearning old and outmoded knowledge. There exists sparse literature regarding the unlearning that takes place at a physician level. Our research objective was to elucidate the experience of trying to abandon an outmoded clinical practice and its relation to learning a new one.

**Methods:**

We used a grounded theory-based qualitative approach to conduct our study. We conducted 30-min in-person interviews with 15 primary care physicians at the Cleveland VA Medical Center and its clinics. We used a semi-structured interview guide to standardize the interviews.

**Results:**

Our two findings include (1) practice change disturbs the status quo equilibrium. Establishing a new equilibrium that incorporates the change may be a struggle; and (2) part of the struggle to establish a new equilibrium incorporating a practice change involves both the “evidence” itself and tensions between evidence and context.

**Conclusions:**

Our findings provide evidence-based support for many of the empirical unlearning models that have been adapted to healthcare. Our findings differ from these empirical models in that they refute the static and unidirectional nature of change that previous models imply. Rather, our findings suggest that clinical practice is in a constant flux of change; each instance of unlearning and learning is merely a punctuation mark in this spectrum of change. We suggest that physician unlearning models be modified to reflect the constantly changing nature of clinical practice and demonstrate that change is a multi-directional process.

**Electronic supplementary material:**

The online version of this article (doi:10.1186/s13012-017-0555-2) contains supplementary material, which is available to authorized users.

## Background

Clinical practice change is difficult. This is illustrated by the proverbial 17-year time lag from idea to practice and the lag in evidence use in practice [1]. Terms such as “creature of habit” and “resistance to change” have been used to describe humans in general and physicians in particular [2–4]. There are many models of physician behavior change but little agreement beyond the difficulty of accomplishing it [5, 6]. A variety of factors have been proposed to account for the difficulty, most focusing on barriers to learning and implementing new knowledge [7, 8]. Consequently, conceptual frameworks of implementation include behavior change at their core [9–11]. The Theoretical Domains Framework is an overarching framework of behavior change which integrates 128 constructs from 33 different theories [12, 13]. The recognition that there is considerable use of low-value care and even zero or negative value care (harm) has prompted national initiatives such as Choosing Wisely [14]. Addressing this issue requires not necessarily learning *new* knowledge, but rather unlearning *old* and outmoded knowledge [4, 15]. Many terms in addition to unlearning have been used to describe this process, e.g., de-implementation, de-adoption, and de-diffusion [16, 17]. Although physicians have a difficult time adopting new practices, they seem to have an even more difficult time “unlearning” outmoded ones [4].

Unlearning, which can occur at the individual and/or the organizational levels, has been defined in many ways, and the nature of unlearning itself is still contested. For example, the phenomenon of extinction describes a form of behavior change in which the strength or rate of a behavior declines when the reinforcing outcome is eliminated such that the behavior goes away, i.e., it is extinguished [18]. Some have considered this to be an example of unlearning while others contend that the original behavior is not erased, but remains, leaving the possibility of relapse [18–20]. Similarly, avoidance behavior and counterconditioning may be relevant to the general phenomenon of unlearning [19, 21, 22]. Notwithstanding these considerations, applied to the management context, Becker defined unlearning as “the process by which individuals and organizations acknowledge and release prior learning (including assumptions and mental frameworks) in order to incorporate new information and behaviors” [23]. Rushmer and Davies distinguished between routine unlearning (and subsequent re-learning) and deep unlearning which requires a substantive break with previous modes of understanding, doing, and being [24].

Most healthcare-related studies of unlearning have usually adapted models of change from the fields of business and management, focusing on identifying various drivers and barriers to the unlearning process faced by healthcare professionals, including those in management positions [17, 23, 25–34]. There have been studies that describe a variety of psychological and other phenomena that might affect a physician’s ability to change practice in general and unlearn specific clinical practices in particular [4, 8, 35–42]. Many of these studies use constructs from the Theoretical Domains Framework. Nevertheless, the topic of unlearning by physicians remains relatively unexplored.

The objective of this study is to elucidate the experience of trying to abandon an outmoded clinical practice and its relation to learning a new one. In so doing, this study aims to better understand the intricacies of individual physician unlearning in order to inform more successful de-implementation strategies for outmoded, ineffective clinical practices.

## Methods

This qualitative study utilized a grounded-theory approach aligned with the development of grounded theory that flows from the transition of Strauss and Corbin and Charmaz, which allows investigators to listen open-mindedly to the experiences of participants in real-life situations and enable “continuous interplay between data collection and analysis” [43, 44]. For quality control, we utilized the consolidated criteria for reporting qualitative research (COREQ) [45].

We obtained our data from interviews and used an iterative process to refine the interview methodology. After conducting an interview, we reflected on which questions were most successful in eliciting responses that reflected the interviewees lived experience and which questions were less generative. Using a constant comparative approach, we made adjustments in our interview method, using theoretical sampling to guide the respondent selection process, seeking data to develop the properties of theoretical categories until the categories had been saturated, i.e., we were not surfacing any new dimensions or properties in subsequent interviews [43].

### Sample

Our study participants consisted of a purposeful and carefully selected group of primary care generalist physicians who practice at a single urban academic tertiary care facility, the Cleveland VA Medical Center and its community-based outpatient clinics. This provided a relatively homogeneous group, as these physicians have similar training backgrounds, clinical experiences, and institutional influences. After interviewing an initial group of five physicians suggested by one of the authors (DCA), we asked each respondent if they could recommend other physicians who would be interested in and qualified to be interviewed for this project. Using this snowball sampling, we continued to interview physicians until we reached the point of theme saturation, which was achieved with a sample size of 15 respondents.

All of the study participants were geographically located in the greater Cleveland area. These participants completed their residency training in Internal Medicine and described their current roles as either “general internists” or “primary care physicians.” The study participants differed in their years of practice post-residency, ranging from 1–30 years, and the proportion of time spent on clinical responsibilities, but most were primarily clinicians (Table 1). These differences in clinical experience helped to generate a collection of thoughtful and unique responses regarding a common set of unlearning experiences. We intentionally chose these respondents, because they are good representatives of the larger group of academic generalist physicians. Woman physicians accounted for 67% of those interviewed.

**Table 1:** Interviewee characteristics and their examples of unlearning

Respondent	Gender	Years post-residency	Practice breakdown	Unlearning example 1	Unlearning example 2	Unlearning example 3
1	F	13	30% clinical, 40–50% administration, 20–30% teaching	Paper records vs electronic health record	Lovenox treatment	
2	F	10	50% clinical, 50% administration, teaching	Multidrug-resistant strep. aureus (MRSA) treatment	Hyperlipidemia treatment	
3	M	3	100% clinical, teaching	Chronic obstructive pulmonary disease (COPD) exacerbation and steroid use	Diabetes treatment	Septic shock treatment
4	F	20	80–90% clinical, 10–20% administration, teaching	Monitoring liver function tests with statin treatment	Hyperlipidemia treatment	
5	F	16	50% clinical, 50% administration, teaching	Hormone replacement	Hyperlipidemia treatment	
6	F	11	50% clinical, 50% administration, teaching	Neurologic physical examination	Prostate cancer screening	Pain medication
7	M	29	100% clinical	Prostate cancer screening	Medical home model	
8	M	6	50% clinical, 50% administration/teaching	TPA protocol for stroke	Red blood cell transfusion guidelines	
9	F	20	10–20% clinical, 80–90% administration, teaching	Hormone replacement	Physical examination	
10	M	3	75% clinical, teaching, 25% administration	Hypertension guidelines	Paracentesis guidelines	
11	F	9	50% clinical, 50% administration, teaching	Pap smear guidelines	Breast cancer screening	
12	F	11	60% clinical, 20% teaching, 20% administration, research	Breast cancer screening	Gynecological physical examination	
13	F	10	450% clinical, 55% research, administration	Prostate cancer screening	Hyperlipidemia treatment	
14	F	1	100% clinical, teaching	Breast cancer screening	Prostate cancer screening	COPD exacerbation and steroid use
15	M	23	50% clinical, 40% teaching, 10% administration	Cardiovascular stress testing	Cardiovascular physical examination	Prostate cancer screening

### Data collection

Data collection began once we received approval for our study from the LSCDVA Institutional Review Board. We used semi-structured interviews to collect qualitative data for this study. All 15 interviews were conducted one-on-one and in-person between September 2014 and May 2015. To set up such interviews, potential participants were e-mailed with a brief summary of the study, estimated time commitment, and expectations of the participant and asked whether they would like to partake in the study. Upon meeting with the participant, informed consent was obtained. We asked the participant for permission to record the interview and explained how the data would be saved in a safe and de-identified manner. After obtaining permission, we then recorded the entire discussion with a digital recorder.

The interviews were conducted using an interview guide with open-ended questions designed to elicit detailed and thoughtful answers. The interview guide is shown in the Additional file 1. We encouraged participants to speak openly regarding their personal experiences with specific examples of practice change and to reflect upon how they responded to such change. Their examples are shown in Table 1. We followed up each question with probing questions to help guide or re-direct the conversation when necessary. We ended each interview, asking participants for their comments regarding practice in general. This method was successful in engaging the participants and in prompting their discussion of factors that they might not have otherwise spontaneously volunteered. Each interview lasted between 20 and 30 min, consistent with the time period of which participants were informed.

The researcher (DG) who conducted the interviews was a second year medical student at the time. Given her lack of personal clinical experience, she had few preconceived notions regarding physician unlearning and clinical practice change. The same researcher (DG) also transcribed the interviews from the audio recordings taken during each session. We repeated the audio recording once through and compared it with the transcript to ensure accuracy as well as to take notes on the respondent’s emotions and intonation. Transcripts were reviewed by all authors. The audio recordings and transcripts were all de-identified and safely stored on a password-protected computer.

### Data analysis

**Fig. 1:**
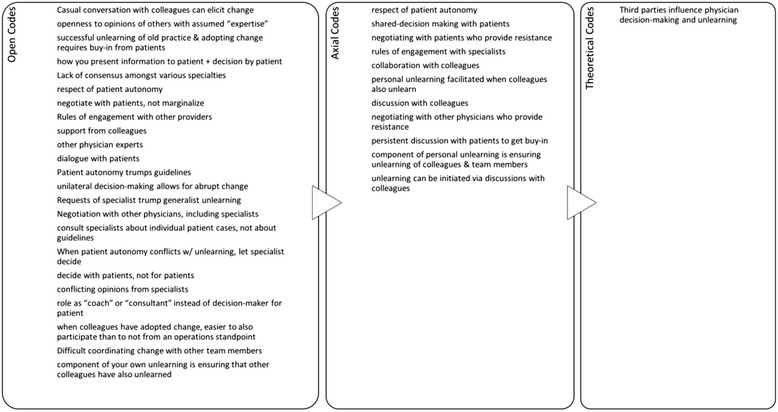
Example of the coding process. The three-step process described by Strauss and Corbin was used to code the data, starting with open codes, followed by axial codes, and ending with theoretical codes

To analyze the data, one team member (DG) generated codes that both summarized and categorized each piece of data in the interview transcripts [43]. The other team members reviewed the coding. Differences were resolved by consensus. These codes assisted with the interpretation of the data and the identification of underlying themes. We used a three-step process described by Strauss and Corbin to code the data, starting with open codes, followed by axial codes, and ending with theoretical codes. We identified a total of 204 open codes, which were distilled into 52 axial codes, which were then further synthesized into 5 theoretical codes (or themes). All three team members applied abductive reasoning to those themes, asking “what core set of elements and relationships are implied by these themes that can explain the unlearning successes and failures that our subjects reported?” Our findings emerged from that multi-stage process. An example of this coding process can be seen in Fig. 1.

Three of the emergent themes ultimately identified by this coding process concern the following: disturbing the equilibrium with practice change, practicing with a purpose vs. cookbook medicine, and questioning “evidence-based medicine” and implications. As part of analyzing our interview data, we engaged in memo-writing [43]. Writing memos helped us to develop our conceptual framework and ensure that our emerging insights were truly grounded in the data. For each respondent, we wrote a memo that touched upon the themes we identified supported by specific data in the transcript that supported our interpretation. This process demonstrated that the themes we identified were supported by all 15 interviews and representative of the entire sample, as opposed to being heavily influenced by a few select interviews.

## Results

### Finding 1: practice change disturbs the status quo equilibrium—establishing a new equilibrium that incorporates the change may be a struggle

When a change is introduced, be it through the introduction of new guidelines or self-initiated change, physicians face various struggles in order to successfully change practice and restore the “equilibrium.” One such set of struggles includes those related to the change itself, such as the ease of implementation or inherent discomfort with the change. Respondent 2, referring to new cholesterol guidelines, describes it as follows:

“Their whole background of thinking – this whole idea of not treating to a number. I think we like to have finite goals and endpoints, which some will argue we still have that, now we’re just treating to a goal dose instead of a goal LDL. I think it’s good to treat a patient and not a number, so that’s fine. It’s still a shift in paradigm. Many changes not only challenge old habits but also require a shift in paradigm regarding a physician’s approach to the practice, resulting in even more of a struggle in adopting the change.”

The nature of the change itself also affects the ease of unlearning. For instance, a change that requires an addition is perceived to be much easier by our respondents to implement than a change that requires the cessation of a current practice. Respondent 3 stated:

“It’s easier to introduce something new than to take out something old. Bringing in a new drug, a new pill to people and telling them that this has showed good evidence, people will introduce it right away, it will be on formulary in 2 weeks and physicians will be prescribing it left and right. Telling people to stop doing something is almost close to impossible. It takes so many years, it takes so many changes, it takes so many events, and it still doesn’t happen in 100% of the people.”

Moreover, changes in guidelines often introduce uncertainty into a field of practice that was very “certain” and well defined in the minds of many physicians. Respondent 2 adds:

“I guess just again thinking you knew something. So I guess that’s the hardest thing is feeling like ‘oh, I thought I mastered my knowledge about something but now I didn’t or now it’s changed’. It just adds a barrier to the patient outcome.”

Another source of struggle that physicians face is a result of disjointed decision making involving third parties, such as hospital management, physician colleagues, and support staff. For instance, a physician might have incorporated the change into his/her practice, but the other staff that the physician works with have not unlearned the current practice. Respondent 4, regarding the new cholesterol guidelines that call for the cessation of regular liver function tests (LFTs) for patients on statins, states:

“What is annoying to me as a clinician is that somebody who runs the clinic thought it would be a good idea for efficient patient care for patients that haven’t been seen in a while to have a nurse go through their record and see if they need labs done prior to their visit with their clinician. I don’t know what the nurse’s guidelines are or why she does it but she goes ahead and blanket orders a CMP (comprehensive metabolic panel), an A1c, I don’t know what prompts her to do whatever she does, LFTs, she’ll get everything. So then the patient gets all this stuff.”

Patients may also provide resistance to unlearning depending on both their understanding of and comfort level with the change. When Respondent 6 decided to change her recommendations regarding NSAID use, she explained, “This was challenging because telling patients that they can’t take ibuprofen doesn’t go over well, but it’s a product of learning new information and assimilating it into practice. Sometimes I get resistance from patients.”

While these opposing forces complicate the unlearning process, physicians that successfully unlearn continually balance these forces, implying that the new equilibrium they arrive at is dynamic in nature. For instance, part of establishing the new “equilibrium” requires physicians to build experience with the new practice to overcome the initial discomfort. As Respondent 4 explains,

“Sometimes it’s not so clear what to do, but I have a pretty high risk population and I’m dealing with a pretty safe drug and I’ve got a lot of years experience using it and reading about it, so I’m relatively comfortable with when and how to use the drug and when not to.”

While guidelines do help to guide practice, they do not instruct on how to implement a practice change in every patient scenario. As a result, successful unlearning requires physicians to not only use their prior experience and clinical judgment but also build new experience with the practice change in a continual fashion to engage in the new equilibrium.

### Finding 2: part of the struggle to establish a new equilibrium incorporating a practice change involves both the “evidence” itself and tensions between evidence and context

While evidence-based medicine is often cited as a driver for change and unlearning, what is considered to constitute evidence varies greatly among providers. When we asked respondents to describe what drives change in their practice, all 15 made references to evidence-based medicine. However, they differed in their interpretations of evidence and the extent to which their practice is truly evidence based. Initially during the interviews, 9 of 15 physicians cited guideline-driven changes as examples of changes in their practice. As physicians had a chance to reflect on their practice, many also came up with examples of non-guideline driven changes—reflecting on their practices and changes in it led to new kinds of changes being recognized. While guidelines are a common example of evidence-based drivers of change, our interviews revealed that there are other sources that provide sufficient evidence to drive change.

First, not all guidelines are created equal and some individuals review the quality of guidelines for themselves [46–48]. Respondent 12 described the process she uses to analyze the validity of guidelines:

“So I had the opportunity to read through all the data that prompted this guideline change. I critically reviewed the evidence myself and convinced myself that this is a guideline I want to follow, given that there are multiple other guidelines out there that other providers are following. I had to convince myself that I believed the evidence and that was the first thing.”

Thus, the quality of the evidence backing guidelines and large studies is an important factor in determining whether physicians will use such evidence to support their unlearning.

When guidelines and randomized controlled trials (RCT) are not available, physicians use local data that they gather from their patient population to support unlearning. Respondent 1 illustrates this using the hesitancy of administering Lovenox without a RCT as an example, “Heparin drips take 12 hours to be therapeutic and Lovenox is therapeutic immediately. So the amount of time that a person spends out of target range on heparin drip is pretty significant yet because there was no evidence, no RCT, people were very reluctant to use Lovenox even though from the common sense standpoint you could easily measure time within target range which was always 100% for Lovenox and something under 100% for heparin drip.” These personal deductions that Respondent 1 made regarding the efficacy of Lovenox were grounded in critical evaluations of patients on heparin versus Lovenox. Respondent 1 used her judgment rooted in clinical expertise to change her practice and provide what she thought to be the best care for her patients. In addition, Respondent 1 had access to local patient data to support her decision. “The pharmacists had done this excellent study looking at our patients at this VA, all of them that had been on heparin drips, and what percentage of time they fell outside the therapeutic range and it was highly disturbing. So that kind of put the nail on the coffin for me.”

Besides clinical observations, there are many other unstructured sources of evidence that guide practice change, such as casual conversations and discussions with colleagues. Respondent 13 describes:

“I think being in an academic environment makes adapting change easier. I think you have local experts that you trust, you have an opportunity to discuss the pros and cons, it’s hard to talk to a paper, and I think that’s an advantage to being in an academic center is having colleagues that are similarly interested in providing evidence-based medicine, you’ve got sub-specialists that similarly do that.”

Without input from colleagues, physicians find it difficult to change their practice solely based off the literature—the opinions of different physicians and discussions on how to incorporate data into practice are critical forms of evidence that physicians use to support their unlearning.

In addition, the respondents differed regarding their opinions of the extent to which it is possible to practice purely evidence-based medicine. Respondent 5, who is at one end of the evidence-based medicine spectrum, argues,

“How you take up a revised recommendation depends heavily on your personal experience and your personal biases, how you interpret literature. And I think this is how it works for everybody, we all have a mouthful with evidence-based medicine, but very few of us are truly evidence-based. Most of us are like a snippit-based, based off of what you hear, what you read, what other people explain to you, what a mentor or somebody you really respect says. It’s a mixture.”

Respondent 5 revealed that a physician’s clinical practice in reality is affected by many sources of information, including classical evidence consisting of published literature, but also unstructured sources of data, such as conversations with colleagues and personal clinical experience. All of these inputs assist physicians in making clinical decisions that are both grounded in data and practical experience.

While every physician we spoke with attested to practicing evidence-based medicine, evidence was very rarely the only determining factor of a physician’s clinical decision-making process. Many physicians stated they used guidelines to “guide” but not necessarily dictate their clinical decisions. Respondent 4 exclaims,

“So certainly anyone with a history of LFT abnormalities, chronic liver disease of any kind, obviously the guideline doesn’t apply to them – if they’re on a statin, I keep monitoring, as being on an additional potential hepatotoxin is a concern. The guidelines are just for patients that have healthy livers and who have no other issues going on, they’re otherwise doing well, no other concern that they’re developing any illness that might involve the liver. I individualize the guidelines. The guidelines don’t say don’t order LFTs, they say only don’t order them for folks who are healthy and who don’t have baseline liver disease. You individualize, obviously we are physicians, we are not robots that just blindly follow guidelines.”

As Respondent 4 demonstrated, physicians individualize guidelines and ascertain whether the guidelines are applicable to a particular patient.

Physicians also approach guidelines with caution because the study population in papers that support guidelines do not always match the much smaller sample of patients that physicians see in their clinics. Respondent 6 expanded upon this discrepancy regarding prostate-specific antigen (PSA) screening guidelines,

“I had a lovely patient, but he does everything I say and he went under my influence and we didn’t get a PSA. When he was 70 he started having back pain and dizziness and he walked into an outside ER, they did a PSA and it was over 100 and he had metastatic prostate cancer and he lived for 6 months or a year, not a long time. I went to visit him in his nursing home and he’d gone down hill. It just killed me. I kept thinking if I had done the PSA maybe I could’ve saved him. So after that, evidence-based medicine was out the window and I’ll sneak in PSA into people’s blood work and not even tell them. I’m exaggerating a little but I’d say let’s get a prostate test today, so they’d say yes so I’d throw it in there. I would rather live with causing biopsy problems or a surgical complication than to have someone die because I didn’t check. For this patient, he was 70 and he wasn’t even supposed to get a PSA but he was complaining about back pain so he got one. But these guidelines are guidelines and they don’t always fit every person and you’re going to order a lot more labs than you really should because of things like this. Something in me changed where there’s a responsibility in taking care of people and I was asking myself what can I live with.”

Respondent 6’s poignant story sheds light upon the disconnection between PSA screening guidelines and the actual patients that Respondent 6 sees in her practice who might actually benefit from PSA screening. On a similar note, Respondent 5 added,

“In the end, on a population level, the heavy use of HRT probably did cause some problems, but for me as an individual I probably didn’t have a single patient in my practice who got breast cancer from HRT, right. So this is sort of the disconnect you have between population management and the few 100 patients I saw.”

Since Respondent 5 had a hard time relating to the guidelines, she left the ultimate decision-making to her patients. Respondent 5 described,

“And I think the key really is that you have to decide with your patient, not for your patient. And I had women that said I’m not coming off HRT, so I said ok you are not coming off HRT.”

Respondent 5’s lack of personal experience with the adverse effects of HRT and respect for patient autonomy drove her to continue prescribing hormone replacement therapy (HRT) for patients that desired it.

The care that the respondents have for their patients was best elicited during discussions regarding the implementation of practice change. All 15 physicians were willing to make exceptions to the guidelines for the sake of respecting patient autonomy and providing patients with care that catered to their needs. Respondent 13 describes,

“Where I have some issues is the moderate versus high intensity statin especially when it comes to side effects for the patients. In someone that is naïve to statins, putting them on a high intensity makes me a little nervous. I think patient factors are important, what’s their adherence like, what is their tolerance in general to medications and side effect profiles. I sometimes err on the side of moderate intensity because for a lot of my patients, if they develop side effects, they’re afraid, and they want to do nothing. I’m looking at the long term, I don’t want him to take the statin for 3 days, I want him to take it for potentially years.”

While the guidelines might have indicated a high-intensity statin for a particular patient, Respondent 13’s care for her patients and concern for possible side effects and long-term medication compliance led to her incomplete adherence to the guidelines.

Other respondents developed similar contingency theories where they presented various patient-specific scenarios that would cause them to be non-compliant with guidelines and use clinical judgment to guide decision-making. Respondent 14 elaborates,

“So I had one person who was like I don’t care I just want the mammogram, because people get worried about cancer. It’s hard to explain harm from screening to patients. I felt like most of them still wanted it. I felt like I tried to explain it but cancer scares a lot of people and a lot of people think ‘I rather go through all this and not have cancer than to have cancer and not know’. I am still trying to respect my patients’ autonomy.”

In these situations, both Respondent 14 and the other respondents’ inherent care for their patients and desire to address their needs outweighed consistent practice change supported by literature-based evidence.

## Discussion

Our findings indicate that practice change disturbs the status quo equilibrium that establishing a new equilibrium that incorporates the change may be a struggle and that part of this struggle involves both unlearning the old evidence itself and tensions between evidence and context. Moreover, this process occurs in the context of the whole system in which physicians practice. Our findings are consistent with some of the models of change that refer to unlearning such as that of Kurt Lewin [29, 49].

Lewin’s change theory provides a linear three-stage model of change, starting with unfreezing of the status quo, change or transition, and finally refreezing. Manchester et al. applied Lewin’s theory to the implementation of evidence-based practice in clinical settings [29]. Our findings support the implication that unlearning, equivalent to the “unfreezing” stage of Lewin’s change theory, is a necessary prerequisite for successful practice change and ultimate “refreezing.” We noted that physicians who develop personal contingency theories and revert back to an outmoded practice to accommodate patient autonomy never fully unlearn or unfreeze the practice, hindering their ability to successfully change their practice. This may be consistent with the dual process model of decision-making, also extended to learning [50–52]. Some physicians may be stuck in a particular heuristic (“fast thinking”) and must change to a more rational approach (“slow thinking”) before the old heuristic is replaced by a new one. Our findings also provide further detail regarding the intricacies of the unfreezing and “change” stages of the change theory, such as the factors that inhibit and support successful implementation of evidence-based practice. For instance, the “Finding 2” section sheds light upon what physicians might consider as evidence-based medicine that initiates practice unfreezing in the clinical setting, ranging from RCTs to local evidence collected by physician observations of patients in the clinic.

An alternative model was developed by Becker in the context of business management [23]. When applied to health care, a modified Becker model of unlearning describes the process of physician unlearning as a product of the tension between prior knowledge—influenced by the prior mental model, characteristics of the clinician, physician’s beliefs about the consequences to patients, social influences, and organization context—and subsequent knowledge [53]. The “prior knowledge” and “tension” phases of the physician unlearning process are in line with the findings from our study. The different struggles physicians face when encountering practice change are manifestations of the tension in the modified Becker model between the previous and subsequent knowledge. Thus, our findings also complement the various factors identified by the model that hinder or help the unlearning process. For instance, in support of the “physician characteristics factor” in the model, our findings elaborated upon the various physician characteristics that might affect the unlearning process, such as personal biases, clinical experiences, and openness to change.

In addition to supporting the modified Becker model, our findings provide further detail regarding factors that both enable and inhibit physician unlearning. For example, not only are physicians’ beliefs about the consequences to patients an important aspect of unlearning, but also the actual resistance from patients and respect of patient autonomy are significant barriers of unlearning. Additionally, physicians’ opinions regarding the actual initiating agent of unlearning, such as the perceived strength and quality of the RCTs or guidelines, influence whether physicians follow through with the unlearning and are not included in the model. As a result, our findings support for the initial phases of unlearning described by the modified Becker model and elaborate upon these phases.

While our findings do support aspects of the modified Becker model and Lewin’s change theory, they are inconsistent with the linearity implied by the two models; the struggle to change is interactive. Thus, “static” models do not capture the dynamic nature of change in the wild; clinical practice is a process rather than a discrete event. When introduced with practice change, physicians often go through a process of trial-and-error with the new practice, developing their own method of implementation and building comfort with the new practice. Very rarely, however, do physicians completely unlearn the outmoded practice. Most physicians develop personalized contingency theories based on particular patient characteristics, as previously demonstrated by Respondent 13’s implementation of statin guidelines and Respondent 14’s respect of patient autonomy when recommending mammogram screening, that guide when they use the new practice and when they revert back to using the outmoded practice. As a result, the unidirectional linear process of change depicted by the Lewin and Becker models does not accurately depict the back-and-forth involved in physician unlearning. The ultimate step of refreezing or development of the subsequent mental model is not fixed; rather, physicians develop a dynamic equilibrium centered around the new practice as opposed to the outmoded practice. As a result, perceived “success” in unlearning may be redefined in this new context, determined by the new equilibrium that is established instead of the degree of relinquishment of the outmoded practice.

Additionally, both the Lewin and Becker empirical models allude to change as an isolated step in the process of transitioning from the prior mental model to the subsequent mental model. These models explain the process of change in the context of stability that the initiating agent disturbs the status quo and the incorporation of the new evidence-based practice into the status quo restores stability or “refreezes” the equilibrium. When physicians change medical practice, however, they find themselves practicing in a new context and new environment, with a new set of expectations and new understandings of what the best practice is. This new equilibrium is plagued with instability and uncertainty as physicians struggle to learn a new practice that they must adapt to their patients with little guidance or direction. The adaption of a new practice, far from restoring stability, introduces further change, as physicians are continually sensing, self-reflecting, discussing with colleagues, critically evaluating, and modifying the new practice. As a result, our findings support a model of medical practice that is a continuous spectrum of change. Each instance of unlearning an outmoded practice and learning a new practice is merely a punctuation in this spectrum of change.

In summary, our findings suggest that current models of physician unlearning be modified to incorporate the constantly changing nature of clinical practice. Such models should demonstrate an episode of unlearning and learning as a snapshot of the constant change that takes place every time physicians carry out a particular practice. Additionally, while unlearning is a necessary prerequisite for learning, rarely does there occur a single unidirectional linear progression from unlearning to learning of a new practice as depicted by both the Lewin and Becker models. Rather, these models should echo a multi-directional process of change, influenced by various structured and unstructured sources of data. This shift in paradigm regarding physician unlearning supported by our findings is also critical to the development of future unlearning models and application of such models to de-implementation strategies.

### Limitations

Our study is based on a non-random sample of 15 respondents who are all generalist physicians practicing at the Cleveland VA Medical Center and its clinics. All of the respondents have experienced instances of unlearning throughout their practices, and many of these instances are similar because they were guideline driven. The respondents’ experiences with unlearning have in some way been affected by their practice at the VA, and many respondents commented during their interviews regarding the effects that practicing at an academic institution and specifically at the VA have had on their unlearning. As a result, we caution against the generalization of this study’s findings to physicians at non-academic institutions and even physicians outside of the VA system. In addition, the authors’ preconceived notions and backgrounds may have influenced the interpretation of the results from this study. In particular, one team member (DCA) is a senior clinician with pre-existing knowledge and familiarity with the literature regarding physician unlearning as well as experience in quality improvement and practice change that may have colored the data analysis; in 35 years of practice, this clinician has experienced multiple instances in which an old practice had to be abandoned because of the new evidence. Some of these were easy, e.g., when the mental model did not require major change, e.g., substituting a better drug for one found to be less effective, and some were more difficult when paradigms changed rather dramatically. Unlearning was required, but the strength of the evidence and its general acceptance won out [54, 55]. Finally, although we describe the phenomenon as “unlearning,” we recognize that not only are there definitional issues that remain but also unlearning itself is a contested issue [56, 57].

### Future research

Given the limitations in our study, we believe future research is warranted to further explore physician unlearning. Since our sample size was limited to 15 generalist physicians within the Cleveland VA system, additional research with a larger sample size in various geographical locations and different practice settings may support the findings in this study or elucidate differences in the unlearning process that occur across various institutions and locations. Additionally, since this study was restricted to generalist physicians only, the findings may not be generalizable to specialist physicians who might approach unlearning differently. As a result, additional studies that examine the unlearning process among various specialty groups are required.

## Conclusions

Our two findings include (1) practice change disturbs the status quo equilibrium. Establishing a new equilibrium that incorporates the change may be a struggle; and (2) part of the struggle to establish a new equilibrium incorporating a practice change involves both the evidence itself and tensions between evidence and context. Our findings provide evidence-based support for many of the empirical unlearning models that have been adapted to healthcare. Our findings differ from these empirical models in that they refute the static and unidirectional nature of change that previous models imply. Rather, our findings suggest that clinical practice is in a constant flux of change; each instance of unlearning and learning is merely a punctuation mark in this spectrum of change. We suggest that physician unlearning models be modified to reflect the constantly changing nature of clinical practice and to demonstrate that change is a multi-directional process.

## Additional file

Additional file 1: Interview guide. (DOCX 16 kb)
