# Supplementary material for: The physician’s experience of changing clinical practice: a struggle to unlearn
Source: Implement Sci. 2017 Feb 28;12:28. doi: 10.1186/s13012-017-0555-2 (PMC5331724; doi:10.1186/s13012-017-0555-2)
Supplement: Additional file 1: — Interview guide. (DOCX 16 kb) [file 13012_2017_555_MOESM1_ESM.docx]

**Interview Guide**

Dr. ___________, thank you for agreeing to be interviewed for this study today.

I would like to start off with a brief introduction of myself: My name is Divya and I am a second year medical student at CWRU School of Medicine. I am working under the guidance of Dr. Aron at the VA on this research project.

*Before we begin, I would like to confirm whether you are willing to have this interview recorded.*

In our discussion today, I would like to focus on changes you have experienced in your clinical practice, and the process you underwent to implement such a change.

Before we begin, I would like to learn a little bit more about you and your practice.

- *How many years have you been in practice?*
- *What specialty are you?*
- *At which clinical site do you spend your most time practicing?*
- *What proportion of your time is spent on your clinical responsibilities (as opposed to research, teaching, administrative responsibilities)?*

Next, can you please describe for me a practice that you have stopped doing or a practice that you have changed?

- *Probe: something that you used to do that you no longer do, something that used to be standard practice, practice should be significant/ change in practice has impact on patient outcomes*
- *Examples: MRIs for lower back pain, new pap smear & pelvic exam guidelines, PSA-based screening, brain imaging studies for syncope, use of epo*

Please describe your experience in making this change

- *Why did you stop the practice?*
- *What initiated this change? How did the change come into your awareness? (national guidelines, hospital management, colleagues, opinion leaders)*
- *What did you consider when deciding to stop doing the practice? (effect on patients, effect on your practice)*
- *How much time transpired when you were considering the change? Stopping the practice?*
- *Can you describe a basic timeline of how you made this change?*
- *How did you stop the practice?*
- *What frustrations/challenges did you encounter during this process?*
- *What/who helped you to stop doing the practice? (attitudes, previous experiences, colleagues, role of staff & management)*
- *What/who made it difficult to stop doing the practice? (attitudes, previous experiences, colleagues, role of staff & management)*
- *Was stopping the practice something you did consciously and intentionally or was it something you found yourself doing?*
- *How did you feel about making this change?*

Can you think of another instance where you stopped doing a practice or changed a practice? Can you share those other examples?

- *Use probes from above*

Is there anything else that you would like to say about your experiences of stopping a certain practice?

*Thank the participant.*
